# Population-Based CD4 Counts in a Rural Area in South Africa with High HIV Prevalence and High Antiretroviral Treatment Coverage

**DOI:** 10.1371/journal.pone.0070126

**Published:** 2013-07-23

**Authors:** Abraham Malaza, Joël Mossong, Till Bärnighausen, Johannes Viljoen, Marie-Louise Newell

**Affiliations:** 1 Africa Centre for Health and Population Studies, University of KwaZulu-Natal, Somkhele, KwaZulu-Natal, South Africa; 2 Department of Global Health and Population, Harvard School of Public Health, Harvard University, Boston, Massachusetts, United States of America; 3 Centre for Paediatric Epidemiology and Biostatistics, University College London Institute of Child Health, London, United Kingdom; Wits Reproductive Health and HIV Institute, South Africa

## Abstract

**Background:**

Little is known about the variability of CD4 counts in the general population of sub-Saharan Africa countries affected by the HIV epidemic. We investigated factors associated with CD4 counts in a rural area in South Africa with high HIV prevalence and high antiretroviral treatment (ART) coverage.

**Methods:**

CD4 counts, health status, body mass index (BMI), demographic characteristics and HIV status were assessed in 4990 adult resident participants of a demographic surveillance in rural KwaZulu-Natal in South Africa; antiretroviral treatment duration was obtained from a linked clinical database. Multivariable regression analysis, overall and stratified by HIV status, was performed with CD4 count levels as outcome.

**Results:**

Median CD4 counts were significantly higher in women than in men overall (714 vs. 630 cells/µl, p<0.0001), both in HIV-uninfected (833 vs. 683 cells/µl, p<0.0001) and HIV-infected adults (384.5 vs. 333 cells/µl, p<0.0001). In multivariable regression analysis, women had 19.4% (95% confidence interval (CI) 16.1–22.9) higher CD4 counts than men, controlling for age, HIV status, urban/rural residence, household wealth, education, BMI, self-reported tuberculosis, high blood pressure, other chronic illnesses and sample processing delay. At ART initiation, HIV-infected adults had 21.7% (95% CI 14.6–28.2) lower CD4 counts than treatment-naive individuals; CD4 counts were estimated to increase by 9.2% (95% CI 6.2–12.4) per year of treatment.

**Conclusions:**

CD4 counts are primarily determined by sex in HIV-uninfected adults, and by sex, age and duration of antiretroviral treatment in HIV-infected adults. Lower CD4 counts at ART initiation in men could be a consequence of lower CD4 cell counts before HIV acquisition.

## Introduction

CD4 counts are important indicators of HIV disease progression [1], [2] and for initiating and monitoring antiretroviral treatment (ART) [3], [4]. Yet, little is known about the variability of CD4 counts in the general population of sub-Saharan Africa countries affected by the HIV epidemic [5]. Studies in sub-Saharan Africa that have investigated CD4 counts either had relatively small sample sizes [6], [7], or used convenience sampling in workers [8]–[13], in reproductive and general health care seekers [12], [14]–[18], in blood donors [14], [19], in HIV counselling and testing attendees [20]–[22], or in healthy volunteers [23], [24]. To our knowledge, the three large population-based studies reporting CD4 data did not aim to evaluate demographic and health factors that might be associated with CD4 cell counts [25]–[27].

In HIV-uninfected individuals, and across populations, CD4 cell counts have been shown to vary with demographic, environmental, immunological and genetic factors [17]. Current exposures to infectious diseases and behavioural factors have also been associated with variations in CD4 cell counts in HIV-uninfected populations [28]. Infections such as pneumonia and tuberculosis (TB) have been associated with decreased CD4 cell counts [29], while higher CD4 counts have been associated with female sex and smoking [8], [17], [30], [31]. Laboratory platform [32] and timing in the day of blood sampling [33] are also known to influence CD4 cell counts. CD4 cell countsIn studies in developed countries, black race, low body mass index (BMI), increasing age and injecting drug use have been associated with lower CD4 counts [30]. Healthy African and Asian populations living in Europe have lower CD4 counts than their European and Caucasian counterparts [11], [28], and significant variations of CD4 cells within African populations have been described [8], [14], [17]. Using data from a population-based demographic and health surveillance in a rural setting in South Africa, we quantify CD4 counts overall and by HIV and treatment status and explore other factors associated with CD4 counts.

## Methods

### Setting and Surveillance

The study took place from May to December 2010, as part of the longitudinal population-based HIV and health surveillance conducted by the Africa Centre for Health and Population Studies in rural uMkhanyakude district of KwaZulu-Natal, South Africa [34]. Individuals are eligible for HIV surveillance if they are reported to be member of a household within a defined geographic demographic surveillance area (DSA) even if non-resident at the time of surveillance. Membership is self-defined on the basis of links to other household members and residency is based on residing at a physical structure within the surveillance area at a particular point in time. For this analysis, the study population consisted of adult (>15 years) residents of the DSA.

### Ethics Statement

Informed written consent was obtained from all adult eligible persons aged 15 year or older for participation in the individual health surveillance and to provide a small blood sample for HIV analysis for research purposes. As permitted by the regulatory framework governing research in South Africa at the time of the study [35], we obtained written informed consent from adolescents aged 15–17 years themselves. Similar to other HIV surveys and surveillance, such as the DHS, the individual health surveillance currently does not reveal HIV results to participants, but instead provides information on location and opening hours of the public-sector HIV counselling and testing facilities, where rapid HIV tests are offered free of charge [36].

We obtained information on ART initiation and duration by linking study participants with the local HIV treatment and care programme database, which is housed at the Africa Centre, and which information can be linked to that of the individual health surveillance at an individual level using a range of variables including surname, first names, date of birth, sex, South African I.D. number, closest clinic, mother’s name and date of death. After linkage, all individual level data were de-identified to prevent analysts working with the data from identifying any of the individuals. Participant names and South African identification numbers are replaced with an anonymous surveillance system number, which analysts cannot link back to the individual participants [37].

Ethical approval for the individual health surveillance (reference BF233/09) and for the linkage between the individual health surveillance and HIV treatment and care programme databases (reference E134/06) was obtained from the Biomedical Research Ethics Committee (BREC) of the University of KwaZulu-Natal, and renewed on an annual basis. The BREC was aware that some of the surveillance participants were minors and approved the age range of participation.

### Sample Collection and Laboratory Methods

Following informed consent, finger prick blood was taken by specifically trained fieldworkers and stored in 2 EDTA-coated 500 µl micro-capillary tubes at room temperature until processing. Samples taken from Monday to Thursday were processed within 24 hours and samples taken from Fridays to Sunday were processed on the following Tuesday. Approximately 60 µl whole blood was aliquoted for CD4 cell count enumeration (FACSCalibur flow cytometer; Becton Dickinson Immunocytometry Systems, San Jose, California, USA). Cells and plasma were harvested from the remaining sample. HIV status was assessed on plasma by enzyme-linked immunosorbent assay (SD BIOLINE HIV 1/2 3.0, Standard Diagnostics Inc., Kyonggi-do, Korea).

### Data Analysis

Medians of CD4 counts between groups were compared using Wilcoxon’s rank-sum test. For regression analyses, we performed a natural logarithm transformation of CD4 counts to remove skewness [31] and make the distribution more normal [38]. We chose logarithmic transformation over square or cubic root transformation, because regression coefficients are easier to interpret.

To investigate factors associated with CD4 counts we performed a multiple linear regression of natural logarithm-transformed CD4 counts against age, sex, HIV status, ART, duration of ART, place of residence (rural, peri-urban, urban), household wealth quintiles, educational attainment, self-reported illnesses (TB, high blood pressure or other serious illness in the last 12 months), body-mass index (BMI) as reported previously [39], and sample processing delay (less than 48 hours for samples collected Monday to Thursday vs. more than 48 hours for samples collected on Friday-Sunday). The multiple regression analysis was repeated after stratifying by HIV status. Duration of ART in years was calculated using the date of ART initiation in the HIV treatment and care programme database and the health surveillance study visit in 2010. Demographic and social data about survey participants were available from the Africa Centre household surveillance [34]. Data analysis and graphics were produced in STATA 11.0 (State Corporation, College Station, TX, USA).

### Comparison with other Studies

To compare our results with those in other sub-Saharan settings, studies of CD4 cell counts in adult populations were identified in PubMed, using the key words “CD4”, “adult”, “HIV”, “survey”, “Africa”. References in relevant publications were checked to identify additional studies.

## Results

**Figure 1:**
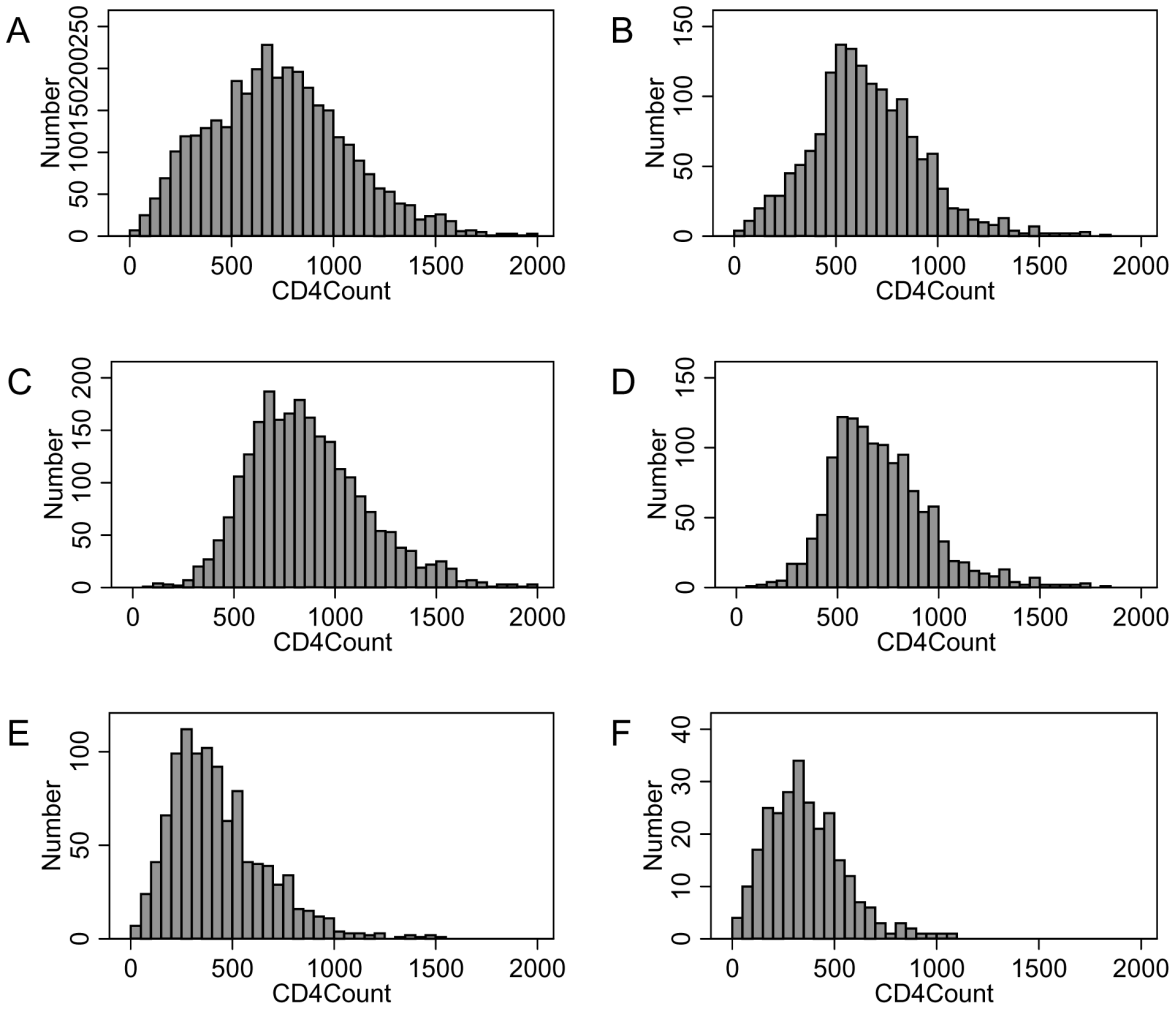
Histogram of CD4 count distributions in the study population by HIV infection status. Total female (A) and male (B) participants; HIV-uninfected female (C) and male (D) participants; HIV-infected female (E) and male (F) participants.

13,253 adult residents were contacted during a home visit and invited to participate in the health surveillance between May and December 2010. Of these, 5990 (45.2%) agreed to provide microcapillary blood samples for HIV antibody and CD4 measurements. A CD4 measurement could not be determined for 982 samples, primarily because of blood clotting (800 samples) and insufficient sample volume (144 samples). A further 15 samples were excluded due to indeterminate HIV ELISA results, leaving a total sample size of 4,993 participants with complete data, of whom 3,432 (69%) were women and 1,561 (31%) men. The median age was 31 years (IQR (inter-quartile range) 20–50), significantly higher (p<0.0001) in women (35 y., IQR 22–52) than in men (23 y., IQR 18–43). Overall prevalence of HIV among participants was 26.2% (1,310/4,993); among female participants, HIV prevalence was30.4% (1,044/3,432) and among male participants, HIV prevalence was 17.0% (266/1,561). The median CD4 count (see Figure 1A and 1B for sex-specific histograms) was 687 cells/µl (IQR 484–904), significantly higher in women than in men (714 vs. 630 cells/µl, p<0.0001). The overall proportion of individuals with a CD4 count below treatment eligibility thresholds of 200 and 350 cells/µl, was 4.2% and 13.5%, respectively.

**Figure 2:**
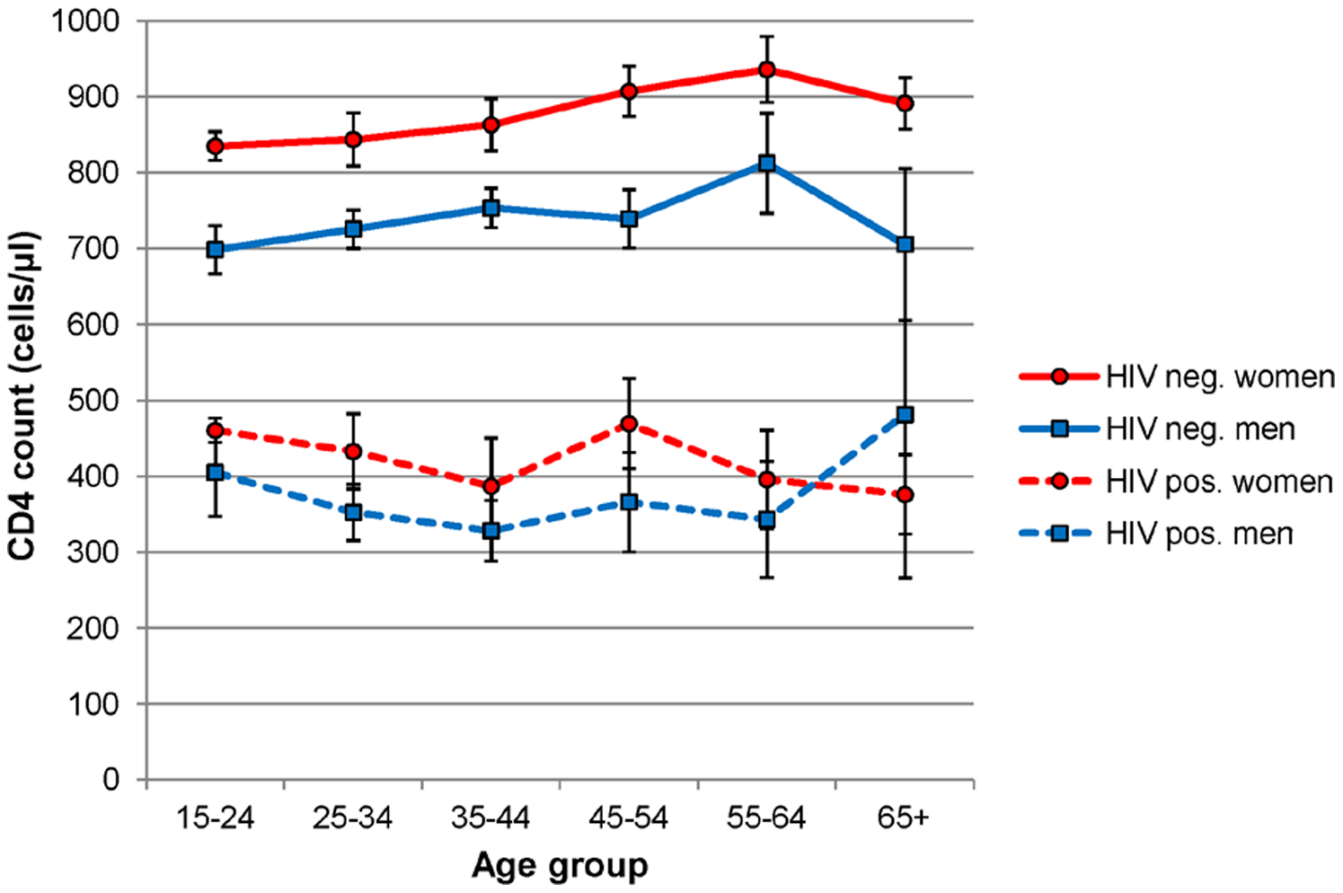
CD4 counts are higher in women than in men after stratifying by HIV status throughout all age groups except in older HIV-infected adults, where sample sizes are too small. Data points represent means and whiskers represent 95% confidence intervals of CD4 counts within HIV-status and age group strata.

The median CD4 count was significantly higher (p<0.0001) in HIV-uninfected individuals (775 cells/µl, IQR 608–974) than in HIV-infected individuals (374 cells/µl, IQR 253–529), for both women (Figure 1C and 1E) and men (Figure 1D and 1F). The difference in CD4 counts between women and men was significant in HIV-uninfected (833 vs. 683 cells/µl, p<0.0001) and HIV-infected adults (384.5 vs. 333 cells/µl, p<0.0001). Figure 2 shows that sex differences were observed throughout almost all age groups and that in HIV-negative persons CD4 counts tended to increase slightly with age. Overall, 14.8% and 45.1% of 1,310 HIV-infected adults had CD4 counts below the treatment eligibility criteria of 200 and 350 cells/µl, respectively. In HIV-uninfected individuals, 0.4% and 2.3% had CD4 counts below 200 and 350 cells/µl, respectively.

**Figure 3:**
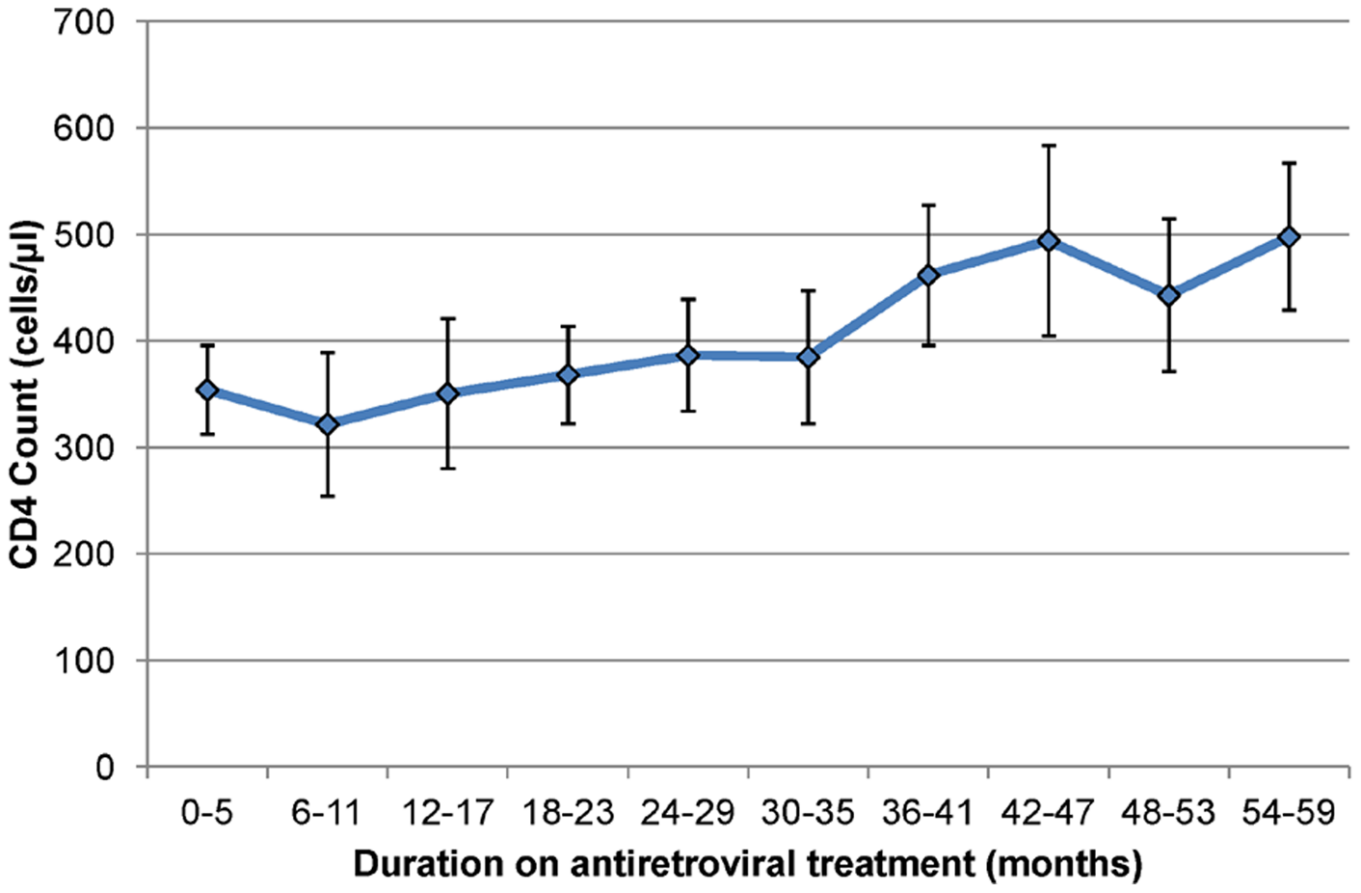
CD4 counts in HIV-positive adults increase after longer durations of antiretroviral treatment. Data points represent mean and whiskers represent 95% confidence intervals of CD4 counts within 6 monthly treatment duration strata.

Among the 1,310 HIV-infected individuals, 391 (29.9%) had been initiated on ART in the HIV Treatment and Care Programme for a median duration of 2.3 years (IQR 1.1–3.5). The median CD4 count in the treated group was 367 cells/µl (IQR, 255–511 cells/µl) compared to 377 cells/µl (IQR, 252–542 cells/µl) in treatment-naive group (p = 0.60). Sex-specific differences in CD4 count remained statistically significant after stratifying by HIV and treatment status (see Table 1). Of the 919 treatment-naive HIV-infected individuals, 137 (14.9%) and 414 (45.0%), had CD4 counts below treatment eligibility thresholds of 200 and 350 cells/µl, respectively. Figure 3 shows that mean CD4 counts in HIV-positive persons on treatment tended to increase almost linearly with longer durations of ART.

**Table 1:** CD4 summary statistics by sex, HIV status and antiretroviral treatment.

	Women			Men			
Category	N	Median	IQR	N	Median	IQR	p-value^1^
HIV-uninfected	2388	833	660–1038	1295	683	542–849	<0.001
HIV+	1044	385	263–546	266	333	214–467	<0.001
HIV+ on ART	323	378	269–525	68	332	208–451	0.004
HIV+ noton ART	721	391	260–561	198	336	226–477	0.001
Total	3432	714	491–948	1561	630	482–813	<0.001

Abbreviations: N (sample size); IQR (interquartile range); HIV+ (HIV-infected); ART (antiretroviral treatment).

1 Wilcoxon rank-sum (Mann-Whitney) test of difference by sex.

In univariate analysis of log-transformed CD4 counts, in line with the large sample size, all examined explanatory variables were significantly associated with CD4 cell counts (Table 2). In multiple regression analysis, sex, HIV infection, ART initiation, ART duration and sample processing delay were all strongly associated with CD4 counts at significance levels of p<0.001. Allowing for age, HIV and ART status, place of residence, household wealth, educational attainment, self-reported diagnosis of chronic diseases and BMI and sample processing delay, women had on average 19.4% (95% confidence interval (CI) 16.1–22.9) higher CD4 counts than men. HIV-infected individuals had on average CD4 counts that were 54.0% (95% CI 52.4–55.6) lower than those of HIV-uninfected individuals. HIV-infected individuals were estimated to have on average 21.7% (95% CI 14.6–28.2) lower CD4 counts at ART initiation (i.e. corresponding to zero years of treatment) than treatment-naive individuals and CD4 count levels increased on average by 9.2% (95% CI 6.2–12.4) with every year of treatment after initiation. Samples collected from Fridays to Sundays (with a processing delay greater than 48 hours) which account for 17.5% of all samples collected had 11% (95% CI 8.2–13.8) lower CD4 counts than samples collected from Mondays to Thursdays (which were processed the day after collection). Excluding these samples from the analysis had no effect on coefficient estimates of explanatory variables (data not shown).

**Table 2:** Univariate and multiple regression coefficients (standard errors) relating natural log-transformed CD4 counts to explanatory variables (N = 4993).

Category	Subcategory	N (%)	Univariate	p-value^1^	Multiple	p-value^1^
Sex	male	1,561 (31%)	Ref.	<0.001	Ref.	<0.001
	female	3432 (69%)	0.089 (0.017)***		0.177 (0.015)***	
Age group	15–24	1,905 (38%)	Ref.	<0.001	Ref.	0.002
	25–34	808 (16%)	−0.298 (0.023)***		−0.02 (0.02)	
	35–44	637 (13%)	−0.301 (0.025)***		−0.075 (0.023)***	
	45–54	649 (13%)	−0.055 (0.025)*		0.007 (0.024)	
	55–64	438 (9%)	0.079 (0.029)**		0.006 (0.027)	
	65+	556 (11%)	0.128 (0.026)***		−0.048 (0.027)	
HIV infection	HIV-negative	3,683 (74%)	Ref.	<0.001	Ref.	<0.001
	HIV-positive	1,310 (26%)	−0.769 (0.014) ***		−0.777 (0.018)***	
ART^2^	no	919 (70%)	Ref.	<0.001	Ref.	<0.001
	yes	391 (30%)	−0.023 (0.037)		−0.245 (0.044)***	
ART duration (per year)^3^			0.083 (0.019)***	<0.001	0.089 (0.015)***	<0.001
Place of residence	rural	2,647 (53%)	Ref.	<0.001	Ref.	0.15
	peri-urban	2,005 (40%)	−0.128 (0.016) ***		0.0 (0.014)	
	urban	341 (7%)	−0.286 (0.032) ***		0.049 (0.027)	
Wealth index	poorest quintile	771 (15%)	Ref.	<0.001		0.779
	2^nd^ quintile	889 (18%)	−0.053 (0.028)		−0.007 (0.22)	
	3^rd^ quintile	958 (19%)	−0.081 (0.027)**		−0.009 (0.022)	
	4^th^ quintile	884 (18%)	−0.123 (0.028)***		−0.02 (0.023)	
	richest quintile	619 (12%)	−0.115 (0.03)***		−0.035 (0.026)	
	unknown	872 (17%)	−0.097 (0.028)***		−0.023 (0.026)	
Education	None	601 (12%)	Ref.	<0.001	Ref.	0.045
	primary	357 (7%)	−0.056 (0.037)		0.004 (0.03)	
	higher primary	538 (11%)	−0.111 (0.033)***		−0.028 (0.028)	
	high school	2,178 (44%)	−0.149 (0.026)***		−0.048 (0.025)*	
	tertiary	195 (4%)	−0.308 (0.046)***		−0.112 (0.039)**	
	unknown	1,124 (23%)	−0.16 (0.028)***		−0.017 (0.027)	
TB	no	4,796 (96%)	Ref.	<0.001		0.481
	yes	197 (4%)	−0.382 (0.04)***		−0.023 (0.033)	
High blood pressure	no	4,163 (83%)	Ref.	<0.001		0.651
	yes	830 (17%)	0.161 (0.021)***		0.009 (0.02)	
Other chronic diseases	no	4,482 (90%)	Ref.	<0.001		0.034
	yes	511 (10%)	−0.127 (0.026)***		0.046 (0.022)*	
BMI	normal	2,085 (42%)	Ref.	<0.001		0.01
	overweight	817 (16%)	0.009 (0.023)		0.007 (0.019)	
	obese	858 (17%)	0.151 (0.023)***		0.054 (0.02)**	
	unknown	1,233 (25%)	0.071 (0.02)***		0.042 (0.016)**	
Sample processing delay	within 2 days	4,120 (83%)	Ref.	<0.001		<0.001
	2 days or more	873 (17%)	−0.12 (0.021)***		−0.117 (0.016)***	

Abbreviations: Ref. – reference, ***p<0.001, **p<0.01, *p<0.05.

1 p-values for variables with multiple categories correspond to a Wald-test that all coefficients of subcategories are zero.

2 Univariate analysis restricted to persons who are HIV-infected.

3 Univariate analysis restricted to persons who are on treatment.

Although the association of CD4 counts with persons aged 35–44, higher educational attainment and self-reported chronic disease was statistically significant, an analysis stratified by HIV status shows these factors to be significant for HIV-infected adults only (see Table 3). Place of residence, household wealth, self-reported TB, high blood pressure diagnosis in the past year and obesity were not independently associated with CD4 cell counts in either HIV-infected and HIV-uninfected adults. Stratifying by HIV status did not modify the effect of sex, but did modify the effect of age on CD4 cell counts. In HIV-negative adults, CD4 cell counts increased gradually up to age 64, whereas in HIV-positive adults, the association with age is more complicated, possibly reflecting differing age-related patterns of HIV acquisition and of ART uptake and retention.

**Table 3:** Multiple regression coefficients (standard errors) relating natural log-transformed CD4 counts to explanatory variables after stratification by HIV infection status.

Category	Subcategory	HIV-negative (n = 3683)	p-value^1^	HIV-positive (n = 1310)	p-value^1^
Sex	Male	Ref.	<0.001	Ref.	<0.001
	Female	0.172 (0.014)***		0.168 (0.044)***	
Age group	15–24	Ref.	0.009	Ref.	<0.001
	25–34	0.003 (0.021)		−0.142 (0.049)**	
	35–44	0.019 (0.024)		−0.276 (0.055)***	
	45–54	0.04 (0.023)		−0.124 (0.066)	
	55–64	0.071 (0.025)**		−0.280 (0.09)***	
	65+	−0.013 (0.024)		−0.11 (0.153)	
ART	no	–		Ref.	<0.001
	yes	–		−0.248 (0.062)***	
ART duration (per year)		–		0.1 (0.02)***	<0.001
Place of residence	rural	Ref.	0.117	Ref.	0.060
	peri-urban	−0.024 (0.014)		0.071 (0.039)	
	urban	0.021 (0.031)		0.121 (0.055)*	
Wealth index	poorest quintile	Ref.	0.247		0.417
	2^nd^ quintile	−0.013 (0.02)		0.007 (0.066)	
	3^rd^ quintile	−0.005 (0.021)		−0.031 (0.065)	
	4^th^ quintile	0.01 (0.022)		−0.096 (0.066)	
	richest quintile	−0.044 (0.024)		−0.007 (0.074)	
	unknown	−0.003 (0.025)		−0.076 (0.074)	
Education	none	Ref.	0.707	Ref.	0.025
	primary	0.01 (0.028)		−0.012 (0.087)	
	higher primary	−0.02 (0.026)		−0.008 (0.08)	
	high school	−0.022 (0.024)		−0.071 (0.071)	
	tertiary	−0.021 (0.039)		−0.262 (0.098)**	
	unknown	−0.032 (0.025)		0.033 (0.076)	
TB	no	Ref.	0.420		0.606
	yes	−0.036 (0.045)		−0.029 (0.057)	
High blood pressure	no	Ref.	0.158		0.337
	yes	0.026 (0.018)		−0.055 (0.057)	
Other chronic diseases	no	Ref.	0.780		0.006
	yes	−0.007 (0.024)		0.128 (0.046)**	
BMI	normal	Ref.	0.026		0.255
	overweight	−0.006 (0.019)		0.025 (0.046)	
	obese	0.033 (0.02)		0.104 (0.053)	
	unknown	0.038 (0.015)		0.048 (0.044)	
Sample processing delay	within 2 days	Ref.	<0.001		0.050
	2 days or more	−0.122 (0.016)***		−0.087 (0.044)*	

Abbreviations: Ref. – reference, ***p<0.001, **p<0.01, *p<0.05.

1 p-values for variables with multiple categories correspond to a Wald-test that all subcategory coefficients are zero.

## Discussion

This study is the first to describe population-based estimates of CD4 distributions in a rural, high HIV prevalence setting where nearly a third of HIV-infected adults are on antiviral treatment. Overall, the median CD4 count of 687 cells/µl in this population was relatively low, which is not surprising given the high HIV prevalence (26.1%) in our study population. The median CD4 count of 775 cells/µl in HIV-negative adults was comparable to that observed in other sub-Saharan countries (see Table 4), although there is substantial variance in reported average CD4 cell counts.

**Table 4:** Median CD4 counts in HIV-negative adults and significant determinants of CD4 counts in sub-Saharan Africa.

Country	Reference	Sample size	Both Sexes	Men	Women	Significant determinants
Ethiopia	Tsegaye [11]	51	660			
Ethiopia	Kassu [9]	780	682^1^	674^1^	749	Sex, study site
Ethiopia	Abuye [8]	1072	695^1^	684	762	Sex, BMI, smoking, study site, khat consumption
Tansania	Ngowi [21]	102	723	597	765	Sex
Botswana	Bussmann [14]	688	726	698	782	Sex, diurnal variation
Ethiopia	Kassa [10]	734	758	713	806	Sex, HIV status, hospitalisation
**South Africa**	**This study**	**4993**	**775**	**683**	**833**	**Sex, HIV status, ART duration, sample processing delay**
Zambia	Kelly [7]	172	780			HIV status
Nigeria	Aina [12]	1291	783^2^	838^2^	818^1, 2^	pregnancy, alcohol, late marriage
Tanzania	Urassa [13]	682	797^1^			
Nigeria	Oladepo [20]	2570	812	746	892	Sex, geographic zone
Malawi	Crampin [49]	214	836	765	911	Sex, location, laboratory platform
Uganda	Lugada [26]	459^3^	836^1^	762^1^	897^1^	Sex
Uganda	Lovvorn [18]	193	838		838	Country
Kenya	Zeh [50]	160	840^1^	811	866	Sex
Senegal	Mair [17]	561	871^1, 2^	712^1, 2^	905^1, 2^	Sex, smoking, high body temperature, late sexual debut
Malawi	Mandala [16]	150^4^	901			
Zimbabwe	Lovvorn [18]	203	912		912	Country
Tanzania	Levin [51]	147	980^2^			
Cameroon	Zekeng [23]	203	980	951	1048	Sex
Guinea Bissau	Lisse [6]	51	1000			
Nigeria	Adoga [25]	1123	1030	935	1121	Sex
Burkina Faso	Klose [15]	186	1082	979	1169	Sex
South Africa	Auvert [27]	930	1128	1057	1180	
Uganda	Tugume [22]	183	1256^2^	1154	1425	Sex
South Africa	Lawrie [24]	719	N.A.			Sex

1 frequency weighted average using subgroups.

2 mean instead of median.

3 participants aged 19+.

4 participants aged 15+.

Abbreviations: N.A. not available.

We found CD4 cell counts to be consistently higher in women than in men, by approximately 20 percent and independent of HIV/ART status, which is consistent with previously published studies reporting higher average CD4 counts in women than in men (see Table 4). In principle, there could be multiple reasons why HIV-infected men have lower CD4 counts than women in HIV-testing and care settings. This includes lower rates of HIV-testing, lower rates of repeat-testing, lower acceptance of linkage to HIV-care after a positive result, and a faster CD4 decline. All of these are conditional on being infected with HIV. However, they cannot explain the observed proportional difference of CD4-levels in HIV-negative women and men after controlling for age and other variables we measured.

In the absence of treatment, HIV disease progression rates have not been reported to differ substantially between men and women [40]. Reports from HIV treatment programmes usually include fewer men than women, with men having a lower CD4 when accessing treatment, which has been taken to suggest that men access treatment at a later stage [41], [42]. However, our results would suggest caution in such interpretation as we show that men tend to have lower CD4 counts than women irrespective of HIV and ART status. This then raises the question whether lower absolute CD4 counts in men have the same meaning as the same CD4 count in women, or whether the absolute level differences are somehow compensated for functionally. Further research is needed to assess CD4 functional capacity at a given absolute level for men and women, the results of which would then further inform the discussion whether HIV treatment eligibility criteria should vary by sex.

We estimated that HIV-infected adults initiating ART had substantially lower CD4 cell counts than treatment-naive HIV infected adults (median 377 cells/µl, IQR 252–542). This finding is expected considering that our study was conducted in 2010, when the eligibility criteria in South Africa were to initiate treatment below a CD4 count threshold of 200 cells/µl in the absence of other criteria. For comparison, from the local Hlabisa HIV treatment and care programme data, median CD4 cell count at first presentation in 2010/11 was 263 cells/µl (IQR 136–444), and median baseline CD4 cell count prior to ART initiation was 145 cells/µl (IQR 76–201) [43]. Although comparisons must be treated with caution because of differing sample collection, transport conditions, storage and laboratory equipment, this finding suggests that, on average, HIV disease progression in our HIV-positive study participants was less advanced than in patients seeking care in clinics.

Our results also mirror to some extent those of another recent study which suggested that a significant proportion of persons initiating HAART under routine conditions in South Africa fail to restore CD4 cell count rapidly despite adequate virologic response [44]. Our study suggests that persons who initiate with low CD4 counts do recover, and that the duration of recovery to levels above the 350 cells/µl threshold could take some time assuming a constant CD4 count recovery per year of treatment. Additional research is clearly warranted to further investigate the dynamics of CD4 cell reconstitution following ART at an individual and population level.

CD4 counts in the current study were lower than those observed in other African populations in Ethiopia, Botswana, Nigeria and Uganda [8], [14], [20], [26], but women in our population had higher CD4 counts than women in Tanzania and both men and women in our study had higher CD4 counts than those reported in a sentinel surveillance cohort in Botswana [14]. This variation across studies and geographical regions may be explained partially by differences in study populations, such as age, ethnicity, the proportion of individuals who smoke and prevalence of underlying diseases, all of which have been shown to be associated with differences in CD4 counts [8], [29], [45]. Differences between studies may be due in part to a lack of adjustment for confounding due to other important risk factors, such as age and cigarette smoking.

Our study had several limitations. First, although study participation levels (45.2%) were high considering that it involves providing a blood sample for research purposes, there was scope for bias. On the one hand, recent work in our setting has shown that young, female and HIV-uninfected adults were more likely to consent to participate in individual health surveillance. Young HIV-uninfected women (who tend to have relatively high CD4 counts) are likely to be somewhat overrepresented in our study nested within individual health surveillance [36]. On the other hand, HIV-infected persons receiving ART or enrolled in pre-ART care were less likely (adjusted odds of 0.75 and 0.62, respectively [36]) than HIV-uninfected persons to participate in individual health surveillance. Moreover, persons receiving ART or enrolled in pre-ART care with CD4 counts ≤200 cells/µl were less likely to participate than persons with CD4 counts >200 cells/µl HIV-infected persons. Thus, persons who have accessed HIV treatment and care and have low CD4 counts are likely to be underrepresented in our sample. For theses, caution should be applied to generalise our findings to other rural populations in Southern Africa.

Second, some of the characteristics (TB, high blood pressure, chronic illness) we evaluated were derived from self report. Although self-reported chronic illnesses have not been validated against clinical assessment in our setting, self-assessments of general health have been found to be strong predictors of short-term but not long-term mortality [46].

Third, data on smoking was not captured in our study, as such we were unable to assess the association of smoking with CD4 counts in this population. In our setting, the prevalence of current smoking in 2003 was substantially higher in men (24%) than in women (2%), such that smoking could potentially be a confounder of the observed sex differences in CD4 cell counts. As smoking is associated with higher CD4 counts in HIV-negative persons [31], sex differences in CD4 counts are likely to be higher among HIV-negative non-smokers.

Finally, our study illustrates that population-based whole blood sample collection in a rural setting has its own challenges. Approximately 13% of collected blood samples could not be tested for CD4 counts due to blood clotting, despite the fact that microcapillaries were coated with anticoagulants. In a logistic regression with clotting as the dependent variable and using the same independent explanatory variables as in our main analysis (i.e. age, sex, location, wealth, education, sample processing delay, TB, high blood pressure, other chronic diseases, BMI), we find that rural (OR 1.96, p<0.001) and peri-urban (OR 1.75, p<0.001) location of the participant home were significantly associated with increased risk of blood clotting after controlling for age and sex, while sample processing delay was not significantly (p = 0.162) associated with clotting. One explanation could be that rural locations are a proxy for longer transport times of samples back to the Africa Centre or for poor road conditions and higher sample agitation. Regardless of the causes of blood clotting, which warrant further investigation, participants from rural and peri-urban areas would thus also be slightly under-represented in our study sample.

Similarly, due to operational reasons related to the rural location, 17.5% of our samples had a delay longer than 48 hours before being processed in the laboratory, which resulted in somewhat lower CD4 measurements. The manufacturer recommendation is to test samples within 48 hours of collection, but it may be longer if blood stabilizers are included in the tube [47]. Our study suggests that population-based CD4 measurements in rural settings can be influenced by both transport conditions and sample storage and processing delays which should be minimized.

In conclusion, we have described for the first time CD4 distributions at a population level in a rural South African setting. Sex, HIV-infection and duration of ART were the most important determinants of CD4 cell counts. Despite high ART coverage in this setting, a large fraction of the population have low CD4 cell counts that put them at increased risk of opportunistic infections like TB [48]. The findings in this study are thus useful for monitoring the impact of the roll-out of ART at a population level, and in particular whether and to what extent CD4 cell counts of HIV-positive persons on treatment have recovered to normal ranges.
